# Mechanisms of Mitochondrial Malfunction in Alzheimer's Disease: New Therapeutic Hope

**DOI:** 10.1155/2022/4759963

**Published:** 2022-05-14

**Authors:** Showkat Ul Nabi, Andleeb Khan, Ehraz Mehmood Siddiqui, Muneeb U. Rehman, Saeed Alshahrani, Azher Arafah, Sidharth Mehan, Rana M. Alsaffar, Athanasios Alexiou, Bairong Shen

**Affiliations:** ^1^Large Animal Diagnostic Laboratory, Department of Clinical Veterinary Medicine, Ethics & Jurisprudence, Division of Veterinary Biochemistry, Faculty of Veterinary Sciences and Animal Husbandry, Sher-e-Kashmir University of Agricultural Sciences and Technology (SKUAST-K), Srinagar J&K-190006, India; ^2^Department of Pharmacology and Toxicology, College of Pharmacy, Jazan University, Jazan, 45142, Saudi Arabia; ^3^Department of Pharmacology, Buddha Institute of Pharmacy, 273209, Gorakhpur, India; ^4^Department of Clinical Pharmacy, College of Pharmacy, King Saud University, Riyadh 11451, Saudi Arabia; ^5^Neuropharmacology Division, Department of Pharmacology, ISF College of Pharmacy, Moga, Punjab, India; ^6^Department of Pharmacology & Toxicology, College of Pharmacy, Prince Sattam Bin Abdulaziz University, P.O. Box-173, Al-Kharj-11942, Saudi Arabia; ^7^Novel Global Community Educational Foundation, Hebersham, NSW, Australia; ^8^AFNP Med Austria, 1010 Wien, Austria; ^9^West China School of Nursing/Institutes for Systems Genetics, Frontiers Science Center for Disease-Related Molecular Network, West China Hospital, Sichuan University, 610041 Chengdu, Sichuan, China

## Abstract

Mitochondria play a critical role in neuron viability or death as it regulates energy metabolism and cell death pathways. They are essential for cellular energy metabolism, reactive oxygen species production, apoptosis, Ca^++^ homeostasis, aging, and regeneration. Mitophagy and mitochondrial dynamics are thus essential processes in the quality control of mitochondria. Improvements in several fundamental features of mitochondrial biology in susceptible neurons of AD brains and the putative underlying mechanisms of such changes have made significant progress. AD's etiology has been reported by mitochondrial malfunction and oxidative damage. According to several recent articles, a continual fusion and fission balance of mitochondria is vital in their normal function maintenance. As a result, the shape and function of mitochondria are inextricably linked. This study examines evidence suggesting that mitochondrial dysfunction plays a significant early impact on AD pathology. Furthermore, the dynamics and roles of mitochondria are discussed with the link between mitochondrial malfunction and autophagy in AD has also been explored. In addition, recent research on mitochondrial dynamics and mitophagy in AD is also discussed in this review. It also goes into how these flaws affect mitochondrial quality control. Furthermore, advanced therapy techniques and lifestyle adjustments that lead to improved management of the dynamics have been demonstrated, hence improving the conditions that contribute to mitochondrial dysfunction in AD.

## 1. Introduction

Alzheimer's disease (AD) is a neurological illness causing progressive cognitive and behavioral deficits. It includes the inability to form recent memories and the loss of previously essential memories. A German neuropathologist named Alois Alzheimer was the first to describe it in 1906 [1]. AD affects both declarative and nondeclarative cognition. Patients have trouble reasoning, understanding intellectual concepts, and even speaking [2, 3]. Early-onset Alzheimer's disease is a rare type of Alzheimer's disease, accounting for about one-two percent of all cases. Mutations in the amyloid-beta precursor protein (APP), presenilin 1 (PS1 or PSEN1), and presenilin 2 (PS2 or PSEN2) loci cause familial AD. Way earlier familial AD is caused by mutations in these genes, which cause a rise in amyloid-(40 or 42) oversupply. The most common form of sporadic AD is late-onset sporadic AD, which appears after the age of 65 and is associated with the APOE4 genotype [4]. A mix of genetic, environmental, and behavioral variables plays a substantial role in late-onset sporadic. Additional polymorphisms may potentially play a role in late-onset Alzheimer's disease [5]. The mitochondrial malfunction has been linked to the development of nearly every neurological illness, including Alzheimer's disease (AD). The relationship between mitochondrial dynamics and amyloid toxicity has been a primary emphasis in this context. According to current research, mitochondrial calcium homeostasis dysfunction is linked to tau and other comorbidities in Alzheimer's disease. Evidence gathered from various models or experimental settings, on the other hand, was not always reliable, which is an ongoing concern in the field. Mitochondria are strategically positioned to play a crucial role in neuronal cell survival or death as integrators of power metabolism and cell death cascades. There is much evidence that mitochondrial malfunction and oxidative damage play a role in Alzheimer's disease etiology. This study examines evidence suggesting that mitochondrial dysfunction plays a significant early impact on AD [6]. Researchers are looking into the link between mitochondrial malfunction and autophagy in Alzheimer's disease. Lipofuscin formation in neurons is caused by insufficient autophagy digestion of oxidatively damaged macromolecules and organelles, worsening neuronal dysfunction. Scientists are particularly interested in developing autophagy-related therapeutics since autophagy is the principal mechanism for breaking down protein complexes and malfunctioning organelles [3]. This review also deals with autophagy as a possible therapeutic target in the genesis of Alzheimer's disease. Dislocation of mitochondria can occur due to interactions with cytoskeleton elements, particularly microtubules, as well as internal mitochondrial processes. It is unclear whether these variances are due to genetic differences. Furthermore, mitochondria in Alzheimer's disease cells are perinuclear, with only a few energetic organelles in the distant processes. They would ordinarily be scattered in healthy cells and essential for synaptic activity, ion channel pumps, exocytosis, and other functions. Elevation in reactive oxidative species and losses in metabolic capabilities characterize AD neurons, and these alterations are visible early in the disease's course. Lower pyruvate dehydrogenase (PDH) protein levels and decreased mitochondrial respiration were seen in the 3xTg-AD brain, indicating mitochondrial dysfunction as early as three months of development. Higher hydrogen peroxide generation and lipid peroxidation were also found in 3xTg-AD animals, indicating increased oxidative stress. At nine months, 3xTg-AD mice had significantly more mitochondrial amyloid-beta (A), which was connected to an increase in A binding to alcohol dehydrogenase (ABAD). Embryonic neurons generated from the hippocampus of 3xTg-AD mice showed a significant reduction in mitochondrial respiration and an elevation in glycolysis. These findings indicate that in the embryonic hippocampus neurons, there is mitochondrial dysfunction which persists in females all through the reproductive period and is aggravated with biological aging. We provide an overview of the fundamental processes that control mitochondrial dynamics and how defects in these pathways accord with the quality control of mitochondria. All these add to the malfunction of mitochondria in AD.

## 2. Mitochondrial Dynamics and Its Functions

**Figure 1:**
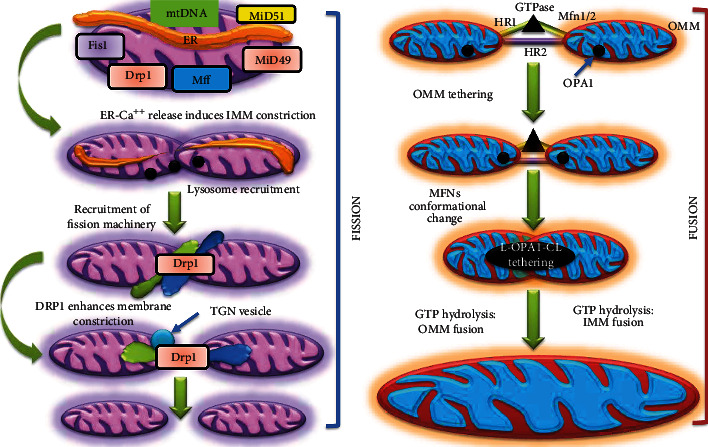
Mitochondrial fission and fusion. Mitochondria are active entities with a constant fission and fusion process that mixes their content. Mitochondrial fusion results in mitochondria that are elongated and extensively linked. For the fission pathway, the main proteins are dynamin-related protein 1 (Drp1), which governs mitochondrial fission in two ways: initially, it is transported from the cytosol to the mitochondrial outer membrane (OM); and secondly, its assembly on the mitochondrial surface causes restriction of the mitochondria, resulting in the division of one mitochondrion into two entities, mitochondrial fission factor (MFF), fission-1 (Fis1), and homologs MiD49 and MiD51. Mitofusins 1 and 2 (MFN1/2) at the outer membrane (OM) and opticatrophy1 (OPA1) at the inner membrane (IM) coordinate mitochondrial fusion, which begins with MFN 1/2-mediated OM fusing of two mitochondria and is accompanied by OPA1-directed IM fusion.

Mitochondria are semiautonomous organelles in cells that carry out a variety of metabolic processes. The mitochondrial structure is very dynamic, switching among both grain-like and thread-like morphology frequently via a process known as fusion and fission, according to recent developments in imaging modalities [4] (Figure 1).

Fusion causes the merging of mitochondria into one, combining cellular contents with mitochondrial DNA (mtDNA) to change into a more resource-rich organelle. Fission leads to mitochondrial reproduction and regulates apoptosis, mitophagy, and alteration in bioenergetic demands.

Fusions can happen when the edges of the organelles clash and merge or when one mitochondrion encounters the edge of another mitochondrion and merge [5]. Mitochondria can fuse after many “efforts,” in which the tip of one mitochondrion slides along the side of another or approaches it many times. Successful fusion is indicated by joint intracellular relocation of the mitochondria accused of being fused [6].

Expanding a mitochondrion can cause mitochondria to fission. The thin region that will become the site of division, on the other hand, may migrate multiple times along the mitochondrion, and sequences of fission and fusion events at the exact location are prevalent [7]. As a result, the dumbbell shape seen in a few large mitochondria appears to be a form that changes slowly. In contrast, more slender mitochondria do not divide into nearly symmetric sections [8]. The mechanisms of mitochondrial fusion and fission and mitochondrial movement are referred to as “mitochondrial dynamics” [9, 10]. Mitochondrial integration in mammals requires the outer mitochondrial membrane-anchored proteins mitofusin1 (MFN1) and mitofusin 2 (MFN2) [11, 12], which incorporate two transmembrane domains linked by a small intermembrane-space loop, a cytosolic N-terminal GTPase domain, and two cytosolic hydrophobic heptad repeat coiled-coil domains. MFN1 and MFN2 can interact in trans with MFN1 on some other mitochondrion to bind neighboring mitochondria or MFN1's coiled-coil domains to form homo- and heterooligomers [13–15]. GTP hydrolysis plays a crucial role in the fusion process. Although the mechanism is unknown, hydrolysis within GTP may cause MFN to shift conformation. Optic atrophy one protein, a dynamin family 100 kD GTPase located either bound to the inner mitochondrial membrane or present in the intermembrane space and is necessary to tether and fuse mitochondrial inner membranes, is needed for mitochondrial fusion [16, 17]. Mgm1p from both mitochondria interacts in trans to anchor and bind the interior membranes after the exterior membranes unite during mitochondrial fusion [16]. By splicing the human OPA1 gene, at least eight mRNA variants of OPA1 are produced [18]. After posttranslational proteolytic processing by mitochondrial processing peptidase (MPP), the longer isoforms are linked to the inner mitochondrial membrane. Still, the S1 and S2 protease site cleavage can give extra short isoforms positioned in the intermembrane gap [19, 20]. The i-AAA protease Yme1 at S2 [21, 22], the presenilin-associated rhomboid-like protein (PARL) at S2 [23], the m-AAA proteases AFG3L2 [15], and paraplegin [24, 25], and the presenilin-associated rhomboid-like protein (PARL) at S2 [21, 22], and the m-AAA mitochondrial membrane potential loss regulates OPA1 cleavage, which devastates the prolonged OPA1 isoform and facilitates S1 separation [20, 22, 25].

Fission in mammalian mitochondria is caused by the combination of dynamin-like protein 1 (DLP1/Drp1, Dnm1p in yeast) and human fission protein 1 (hFis1, Fis1p in yeast). Upregulation of hFis1 or dominant-negative DLP1 or RNAi knockdown of hFis1 or DLP1 enhances the lengthening of mitochondria, implying that hFis1 and DLP1 are the fission proteins required for mitochondrial elongation [26–28]. A single C-terminal transmembrane motif anchors hFis1 in the membrane. At the same time, six helices with two tetratricopeptide repeat- (TPR-) like folds are found in the N-terminal cytosolic region, which is implicated in protein interactions. As per cross-linking and fluorescence resonance energy transfer (FRET), hFis1 and DLP1 connect cooperatively [29]. The 1-helix is involved in the hFis1 TPR's quick contact with a DLP1-fission complex, presumably moderating DLP1–hFis1 couplings. The ability to drive mitochondrial fission is reduced when hFis1 oligomerization-deficient mutants are overexpressed, indicating that hFis1 oligomerization may play a significant role in mitochondrial fission [30, 31].

The number of mitochondria and mitochondrial DNA should be doubled twice during the cell cycle. The nucleocytoplasmic ratio to chondroma in somatic cells is more or less constant [32]. As a result, at the very least, a loose synchronization of reproduction cycles must be expected. Many protists have shown systematic changes in mitochondrial shape during the cell cycle [33]. These changes generally follow this scheme: the chondriome comprises one highly perforated basket-shaped complex that lines the cell's periphery at the start of the interphase [34]. The size of the mitochondrial basket expands during interphase, as does the number of tiny mitochondria. The mitochondrial basket is broken into many fragments during mitosis, which tends to form clusters, and the overall number of mitochondria is significantly decreased again following cytokinesis. The fragmentation of mitochondrial networks into single mitochondria can affect metabolite transport and generate a unique environment for the nucleus [35].

The human membrane-associated RING-CH (MARCH)-5 E3 ubiquitin ligase is located in the outer mitochondrial membrane [36–38]. MARCH5 combines and ubiquitinates hFis1, DLP1, and Mfn2. Mitochondrial elongation is caused by RNAi of MARCH5, showing that mitochondrial fission is prevented. MARCH5 may influence DLP1 trafficking, as abnormal DLP1 clustering has been found in cells expressing MARCH5 RING mutants [36]. MTP18 (Mdm33 in yeast) is located in the inner mitochondrial membrane and faces the intermembrane region [39]. MTP18 expression is activated by phosphatidylinositol (PI) 3-kinase signaling [40]. Mitochondrial fragmentation is caused by MTP18 overexpression, whereas mitochondrial elongation is caused by MTP18 RNAi. The known DLP1-mediated mechanism causes mitochondrial fragmentation when hFis1 is overexpressed. MTP18 knockdown, on the other hand, inhibited this hFis1-induced fragmentation, showing that hFis1 is dependent on MTP18 [39]. The mechanism through which MTP18 contributes to inner mitochondrial membrane fission is unknown.

## 3. Altered Metabolism Due to Dynamic Dysfunction

**Figure 2:**
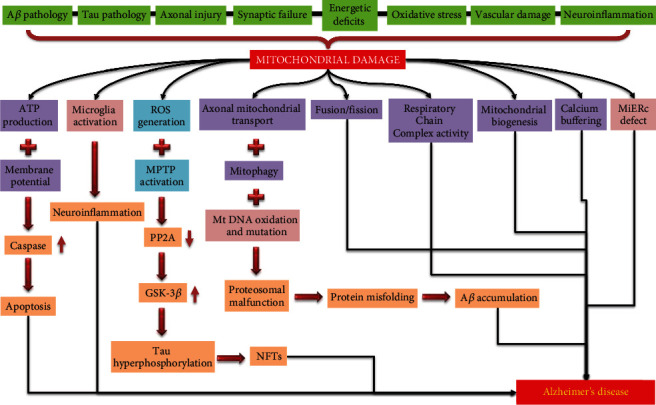
Mitochondrial cascade of neurodegeneration. The reactive oxidative species (ROS) that are inevitably produced during the respiration process build up inside the cell, progressively causing damage to mitochondrial DNA (mtDNA) and associated mitochondrial proteins. An instability in mitochondrial dynamics (i.e., fission/fusion) eventually arises, triggering a negative cycle of increasing ROS production, mitochondrial damage and malfunction, and cell death. The neurodegeneration that is characteristic of Alzheimer's disease (AD) happens as a result of this chain of events.

The etiology of AD has aroused heated debate. Surprisingly, the disease's sick “dots” have begun to be connected through research, revealing the enormously complicated links that cause AD [41, 42]. The extracellular coarse aggregate of amyloid-*β* (A*β*) containing senile plaques has been linked to disease initiation and progression [43, 44]. The microtubule-associated protein tau, on the other hand, is another protein identified as a key participant in AD since its hyperphosphorylated, aggregated fibrils hold neuronal space as neurofibrillary tangles (NFTs) in vulnerable areas of AD brains (i.e., the hippocampus and cortices) [45, 46]. Although much about Alzheimer's disease etiology is still unknown, indications point to mitochondrial dynamics as a likely reason (Figure 2).

Neurons rely on mitochondrial ATP production to create axonal and synaptic membrane potentials and sustain ionic gradients. The oxidative phosphorylation (OXPHOS) activity is strictly regulated by the constant Ca^2+^ levels in the mitochondrial matrix. Unfortunately, if mitochondrial metabolism becomes intoxicated due to excessive Ca^2+^ conversion from the ER or increased cytosolic Ca^2+^, this can increase oxidative stress, obstruct mitochondrial membrane permeabilization, and reduce ATP production, all of which can lead to cell death [47]. Extensive research has accumulated evidence that Ca^2+^dyshomeostasis and mitochondrial dysfunction occur in neurons in the AD brain. As the cell's energetic and energy centers, mitochondria are crucial to cellular proliferation, and defects in mitochondrial dynamics frequently precede many of AD's characteristic diseases [48, 49]. Reduced ATP levels and mitochondrial function with increased ROS production are AD signs [48, 49]. Mitochondrial damage can also be seen in peripheral tissues of Alzheimer's patients. Likewise, mitochondrial dynamics and bioenergetics are disrupted in fibroblasts from Alzheimer's patients and Ca^2+^ dyshomeostasis [50, 51]. Other mitochondrial Ca^2+^-related disturbances, such as mitochondrial Ca^2+^ buffering, mitochondrial dynamics (trafficking, fission, and fusion), and mitophagy, are thought to be altered in AD and are caused by mitochondrial Ca^2+^ dyshomeostasis before cell death [21, 52–58].

**Figure 3:**
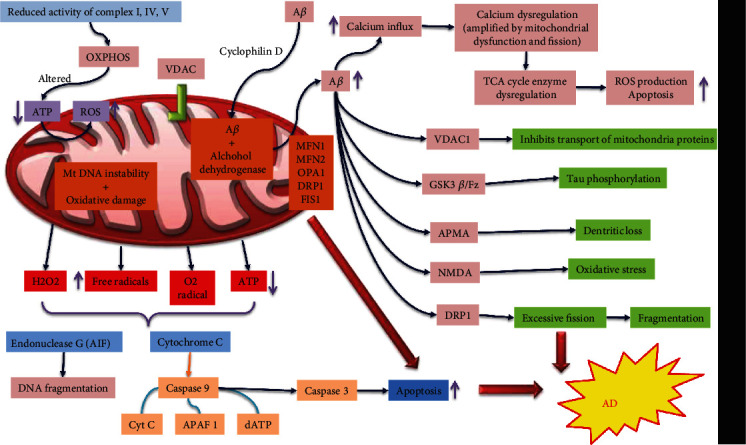
Mitochondrial dysfunction in Alzheimer's disease. Mitochondrial abnormalities linked to increased oxidative stress have long been thought to have a factor in the cell death and deterioration seen in Alzheimer's disease. However, as Alzheimer's disease progresses, mitochondria undergo significant changes that result in decreased ATP production and increased reactive oxygen species production (ROS). Mitochondria also lose their calcium (Ca^++^) buffering capacity, which can set off a chain reaction within the cell that is harmful. When apoptosis is induced, mitochondrial dysfunction releases many proapoptotic molecules. These factors either directly activate apoptosis by associating with cytosolic factors to produce the apoptosome, or they indirectly trigger apoptosis by combining with cytosolic factors to generate the apoptosome. Ultimately, proapoptotic mitochondrial proteins translocate into the nucleus to fragment deoxyribonucleic acid (DNA). Overall, these mitochondrial changes are associated with cell death and deterioration.

Unlike fission/fusion processes, mitochondrial activity is also controlled by the location of organelles inside the cell. The cytoskeleton and associated proteins regulate mitochondrial distribution, ensuring that locations with high metabolic demands receive the most [59, 60]. Interestingly, mitochondrial dynamics impact mitochondrial distribution: both fission alleles with extended mitochondria (such as mutants of DLP-1) and fusion mutants with small, spherical mitochondria (such as OPA-1 mutants) produce alterations in mitochondrial distribution across the cell [61]. More importantly, because neurons have far higher energy demands than any other cell type (such as the extremely energy-intensive operation of ion channels and pumps, signal transduction, axonal/dendritic transit of signal molecules and vesicles, and so on) and rely on mitochondrial integrity [62, 63], this balance is critical for brain function. Any disruptions in mitochondrial function would predispose the neuron to various negative consequences, including the neurodegeneration found in Alzheimer's disease [64] (Figure 3). Late-onset sporadic AD's direct cause(s) is unknown, although age is a significant risk factor [65]. Ca^2+^ dyshomeostasis and mitochondrial dysfunction are found in neurons in Alzheimer's disease, according to years of research. Discovering targets to sustain Ca^2+^ homeostasis and mitochondrial health is also essential as a promising method for avoiding or lowering the pathology that underpins Alzheimer's disease.

### 3.1. Mitochondrial-Mediated Oxidative Stress

Current research indicates that oxidative stress is the principal cause of pathology linked with Alzheimer's disease, and there is substantial evidence to support this claim. An oxidative damage biomarker, 8-hydroxyguanosine (8-OHG), appears decades before A*β* senile plaques appear. At the same time, A*β*PP-mutant Tg2576 transgenic mice demonstrate oxidative damage before A*β* aggregation [66–74]. When A*β* is generated, it is oxidized, and the tyrosine cross-links render the insolubility of peptide and hence more likely to accumulate [75]. A*β* is thus damaging to the cell in its consolidated condition because it increases oxidative stress [76] and damages mitochondrial function. The latter mechanism was revealed in the M17 cells, which are overexpressing mutant A*β*PP (these cells had a four-fold lower rate of mitochondrial fusion [77]); investigations demonstrate that A*β* overexpression creates mitochondrial fragmentation, malfunction, excessive oxidative stress, and decreased ATP synthesis [78]. *In vitro*, cytochrome oxidase, pyruvate dehydrogenase, and ketoglutarate dehydrogenase have been shown to produce reactive oxygen species (ROS), hydrogen peroxide (H_2_O_2_), and superoxide radicals (O^2-^), which are primarily produced in the electron transport chain [79].

Moreover, the reactions that occur inside a neuron guarantee the regular production of a high number of ROS: the average nonneuronal cell uses 10^13^ O_2_ molecules per day in metabolic processes, and around 10^11^ free radical is produced [79]. Synaptic function is harmed when A*β* accumulates at synaptic terminals. A*β* can also induce damage to synaptic mitochondria. Mitochondrial destruction in synaptic mitochondria is anticipated to be larger than in cell-body mitochondria [80]. Damaged synaptic mitochondria may not provide enough energy to synapses, resulting in decreased neurotransmission and, eventually, intellectual failure.

Numerous recent studies have discovered that A*β* binds to mitochondrial proteins such as mitochondrial fission protein Drp1 [81], mitochondrial outer membrane protein VDAC [53], and mitochondrial matrix proteins A*β*-binding alcohol dehydrogenase and CypD [82] and that these anomalous connections cause severe free radical production, mitochondrial fragmentation, and mitochondrial biogenesis, inevitably leading to mitochondrial function. Furthermore, A*β* enhances calcium's ability to enter the cell. The mitochondria then take in the calcium, which is one of the cell's calcium regulators [83]. When high quantities of A interact with VDAC1 and prevent mitochondrial protein transport, mitochondria become dysfunctional. More free radicals are produced due to this malfunction [84]. The creation and clearance of free radicals are out of balance, resulting in oxidative stress, characterized by the synthesis of 8-hydroxyguanosine [61, 85].

Furthermore, due to short-term exposure to ROS, mtDNA mutation causes mitochondrial anomalies in fission and fusion [86]. Mfn1/2-null cells and OPA-1-deficient cells have many fractured mitochondria and a high number of endogenous and disconnected respiratory rates. The latter events are caused by a decrease in electron transport rates in complexes I, III, and IV [13, 87]. Inhibition of DLP-1 results in reduced ATP synthesis due to inefficient oxidative phosphorylation and decreased complex IV activity [88, 89]. Fission/fusion imbalance caused by mtDNA mutations can affect calcium homeostasis. Both abnormal fragmentation and lengthening of mitochondria result in increased ROS production in the cell and excessive iron deposition [90–95].

### 3.2. Low Mitochondrial Bioenergetic Performance

In Alzheimer's disease, there is a widespread shift away from glycolytic energy synthesis toward the use of ketone bodies, an alternative fuel. A 45 percent decrease in cerebral glucose utilization in Alzheimer's brains is accompanied by a decrease in glycolytic enzyme expression and activity of the pyruvate dehydrogenase (PDH) complex [96–98]. The “cybrid model” of Alzheimer's disease has revealed that mitochondrial malfunction plays a role in the disease's progression [99]. COX activity is reduced, the mitochondrial membrane potential is reduced, mitochondrial mobility and motility are reduced, oxidative stress is enhanced, caspase-3 is overactivated, and Ab production is raised in AD cybrid cells [100–102].

Furthermore, offspring of women with Alzheimer's disease have a higher risk of developing the disease, implying human maternal mitochondrial inheritance [103, 104]. The female 3xTg-AD mouse brain was found to match numerous signs of mitochondrial failure reported in human Alzheimer's disease patients, including lower mitochondrial bioenergetics, elevated oxidative stress, and increased mitochondrial amyloid deposition, according to a study [105, 106]. PDH and COX expression and activity were dramatically reduced in 3xTg-AD mitochondria. In primary neurons from 3xTg-AD rats, the switch from oxidative phosphorylation to lactic acid-producing glycolysis revealed decreased mitochondrial efficiency [107]. This unstable metabolic state, along with oxidative stress caused by decreased ETC efficiency, results in poor bioenergetics, which compromises brain function and exacerbates Alzheimer's disease [108–110]. PDH is a rate-limiting enzyme in the mitochondria that converts pyruvate from glycolysis to acetyl-CoA, which then condenses with oxaloacetate to commence the TCA cycle to generate energy. PDH deficiency induces pyruvate buildup, which increases extracellular acidification and speeds up anaerobic metabolism to lactic acid, as seen by an increase in ECAR in 3xTg-AD neurons [111]. In 3xTg-AD neurons, however, reduced PDH activity results in a shortfall in acetyl-CoA and, as a result, lowers OXPHOS action, as seen by a drop in OCR [112]. These findings are back up by previous PET metabolic analyses in people at high risk of Alzheimer's disease (AD), mild cognitive impairment (MCI), or late-onset AD, in which impaired glucose pickup and consumption were identified as one of the earliest symptoms of AD, occurring long before the disease manifested [113–119].

### 3.3. Mechanism of Improper Transportation and Autophagy

One of the harmful alterations in major neurodegenerative disorders is altered mitochondrial transport (MT) [120–123]. The kinesin-1 family (KIF5) is the primary motor that facilitates mitochondrial transport [121, 124]. The KIF5 heavy chain (KHC) has an ATPase-active N-terminal motor domain and a cargo-binding C-terminal tail motif. KIF5 motors can attach to mitochondria owing to adaptor proteins like Milton from Drosophila. Milton binds to the C-terminal tail domain of KIF5 and the mitochondrial OM receptor Miro, acting as a KIF5 motor adapter [125, 126]. In Drosophila, the Milton mutant consistently reduces mitochondrial transport into synapses. Milton orthologues Trak1 and Trak2 have been identified in mammals [127–129]. In cultured hippocampal neurons, Trak2 amplification improves axonal mitochondrial motility [120]. Trak1 depletion or mutant expression, on the other hand, causes mitochondrial transport across axons to decrease [130]. Mammalian Trak1 and Trak2 have one N-terminal KIF5B binding site and two dynein/dynactin binding sites, one at the N-terminus and one at the C-terminus according to a new study [131].

Miro, also known as MIRO, gene mutation inhibits anterograde mitochondrial transit and deprives the number of mitochondria in peripheral synaptic terminals in Drosophila [132]. Miro1 and Miro2 are two isoforms of Miro found in mammals. The Miro1-Trak2 adaptor complex modulates mitochondrial transport [128]. Heavy chains (DHC) serve as the motor area for force production, while intermediate (DIC), light intermediate (DLIC), and light chains (DLC) function in cargo adhesion and motility modulation. Drosophila mitochondria are connected with dynein motors, and changes in DHC impact the speed and length of axonal mitochondria backward transit [124]. Mitophagy is a critical cellular mechanism in mitochondrial quality control since it is specialized autophagy to eliminate defective mitochondria. It entails siphoning damaged mitochondria in autophagosomes after that degraded within lysosomes. According to new research, PINK1/Parkin-mediated mitophagy protects mitochondrial viability and efficiency [133–135]. The gradual accumulation of PTEN-induced putative kinase protein 1 (PINK1) on the surface of wounded mitochondria precedes Parkin translocation from the cytosol to the mitochondria in this kind of mitophagy. Other mitophagy mechanisms that are not dependent on PINK1/Parkin have also been discovered. For example, the BCL-2 homology 3- (BH3-) containing peptide NIP3-like X (NIX, also known as BNIP3L), a mitochondrial OM protein, has been demonstrated to play a vital role in the removal of mitochondria in erythrocytes [136]. On exclusion membranes, NIX has an amino-terminal LC3-interacting region (LIR) that connects to LC3 [137]. In erythroid cells, this allows NIX to function as a unique mitophagy receptor, physically binding the autophagy machinery to the mitochondrial surface. Mitophagy must be studied using a variety of complementing assays because it is such a dynamic system. These tests must be supplemented with the use of drugs that disperse mitochondrial membrane potential and a flux inhibitor to trap newly generated autophagosomes [138–140]. Distended patches with aberrant numbers of organelles (including mitochondria) proliferate in axonal degeneration in Alzheimer's patients [141]. PS1 mutations have been demonstrated to impact kinesin-1-based axonal transport by increasing GSK3 activity and phosphorylation of kinesin-1 light chains (KLC). This defect reduces the frequency of APP, synaptic vesicles, and mitochondria in the neuronal processes of hippocampal neurons and sciatic nerves of mutant PS1 knock-in mice [142]. When established neurons are exposed to A or ADDLs, their mitochondrial activity and frequency in axons substantially decrease [143–145]. In Drosophila, overexpression of Ab slowed bidirectional axonal mitochondria transmission and impoverished presynaptic mitochondria, resulting in presynaptic malfunction [146]. Reduced anterograde transit of axonal mitochondria, mitochondrial dysfunction, and synaptic deficit were all observed in developing neurons from APP Tg mice, all of which could be attributed to oligomeric Ab accumulation in mitochondria [147]. Induction of mitophagy is linked to changes in mitochondrial mobility. Mitophagy is complemented by diminished anterograde mitochondrial transit due to Parkin-mediated Miro degradation [148–151]. According to a new analysis, Parkin-mediated mitophagy is substantially increased in AD neurons of murine models and human brains. As a result, the anterograde transport of axonal mitochondria is decreased in these neurons [147, 152].

For neuronal homeostasis, bidirectional transfer of intracellular components between distal neurites and the cell soma is essential. Autophagosomes and endosomes that combine in the distal axon must be retrogradely delivered to the soma in neurons to fuse with lysosomes and digest their contents [153, 154]. As a result, even minor abnormalities in autophagosome formation, maturation, or trafficking are expected to have disastrous effects on autophagic transit and neuronal homeostasis. Nevertheless, the proof is growing, suggesting autophagic processes are critical for brain health maintenance, especially in degenerative disorders [155]. Surplus or defective mitochondria can be preferentially removed in Saccharomyces cerevisiae, and Uth1 and Aup1 are implicated in this mechanism [156–158]. Kanki and Klionsky [159] recently discovered that ATG11, a gene previously thought to be required exclusively for competitive autophagy, is also required for mitophagy. Mitophagy is also inhibited even under acute famine situations if the carbon source makes mitochondria necessary for metabolism [159]. These findings show that mitochondrial disintegration is a carefully controlled process and that these organelles are mainly immune to generic autophagic breakdown.

Several mitochondria grow and structurally disorganize as the brain ages, while lysosomes eventually amass the nondegradable polymer lipofuscin. The mitochondrial-lysosomal axis theory of aging was proposed by Terman and Brunk [154], under which mitochondrial attrition begins to decline with age, resulting in increased oxidative stress, accrual of damaged organelles and lipofuscin, reduced ATP production, discharge of apoptotic factors, and, ultimately, cell death. Nixon et al. discovered autophagosomes and other prelysosomal autophagic vacuoles in AD brains [160], especially within neuritic pathways. Autophagosomes, multivesicular bodies, multilamellar bodies, and cathepsin-containing autophagolysosomes were the most common organelles in dystrophic neurites. Autophagy was seen in the perikarya of afflicted neurons, especially in those with neurofibrillary disease, which was linked to a reduction in mitochondria and other organelles [160]. As a result, it was discovered that autophagocytosis of mitochondria is common in Alzheimer's disease [161, 162]. Overall, the findings back with a previous study that found a large increase in mtDNA in neuronal cytoplasm and vacuoles associated with lipofuscin in neurons with greater oxidative damage in Alzheimer's disease [68, 163, 164]. COX-1, which is a mitochondrial protein, was shown to be elevated in the cytosol and linked with mitochondria undergoing phagocytosis, as also reported previously [161]. Overall, these findings support the idea that susceptible AD neurons have a high amount of products of mitochondrial degradation, implying either decreased proteolytic turnover rate or increased mitochondrial turnover by autophagy, resulting in accrual of mitochondrial degradation products. Recently, it was discovered that autophagic vacuoles contain A*β*PP and secretase, which contribute to the accumulation of A*β*. They are especially rich in *γ*-secretase enzymatic activity and *γ*-secretase complex components [165, 166]. These findings show that accumulating autophagic vacuoles in dystrophic neurites may also promote the local synthesis of A*β* within plaques. The neuropil's widespread surge in autophagy may be a substantial source of A*β* overproduction in the AD brain. Breakdown and spillage from postlysosomal vesicles cause cytosolic acidification, other membrane and organelle damage, and eruptive cytoplasm degradation, all of which contribute to neuron demise.

### 3.4. Defective Mitophagy

Defective mitophagy leads to the accumulation of dysfunctional mitoplast, which leads to the progression of AD. To ensure effective mitophagy, autophagosome containing mitoplast should fuse with a lysosome, and lysosome should cause digestion of these organelles. Recently, it has been found that neurons of patients affected with AD exhibit abnormal accumulation of autophagosomal vacuoles. On further investigation, it was observed that accumulation of autophagosomal vacuoles occurs due to lysosomal dysfunction and defective fusion between autophagosome and lysosome [167]. The sirtuins (SIRTs) are the enzymes that are involved in the prevention of various age-related diseases, including AD. Of the seven isoforms of SIRTs, SIRT-I and SIRT-III play an important role in mitophagy of defective mitoplast through deacetylation/activation of prominent mitophagic proteins. Recent studies have found significantly reduced levels of SIRT-I and SIRT-III (which leads to the accumulation of defective mitoplasts) in cortical regions of the brain of patients with AD [168].

Furthermore, in recent times, NAD^+^ has been found to regulate the delicate balance between biogenesis and mitophagy of mitochondrion. Decreases in NAD^+^ lead to the accumulation of defective mitoplasts. In AD, decreased levels of NAD^+^ have been attributed to the activation of several NAD^+^ consuming classes of enzymes. Furthermore, NAD^+^ constitutes a cofactor of several enzymes involved in the protection of DNA damage; for instance, poly(ADP-ribose) polymerase 1 (PARP1), cyclic ADP ribose hydrolase (CD38), and CD157 classes of enzymes are actively involved in repairing mito DNA damage. Interestingly, any stressful condition in the mitochondrial microenvironment leads to consumption of NAD^+^ as this gets utilized for the synthesis of the above-mentioned enzymes, which ultimately leads to lowering of SIRT-I and SIRT-III activity henceforth promoting amyloidogenesis (180, 181).

## 4. Therapeutic Strategies for the Improvement of Mitochondrial Dynamics

Although numerous elements in disease development have been identified, the intricacies that underpin cognitive impairment and neurodegenerative aspects of Alzheimer's disease are still unknown, of which mitochondrial dysfunction appears to be particularly essential in the development and pathophysiology of AD. Henceforth, researchers worldwide have postulated that effective targeting of these mitochondrial dysfunctions can provide a window in developing novel potential therapeutic strategies for controlling and treating AD and closely associated neurodegenerative diseases. Indeed, researchers have focused on various therapeutic approaches that revolve around mitochondrial repair and effective targeting of mitochondrial antioxidant pathways to check the neurodegenerative cascade. In addition to this, more recently, based on the results obtained from postmortem examination of neurons obtained from geriatric AD patients and animal models, various therapeutic protocols were proposed by clinicians and researchers, which include immunotherapy [169–171], cholinergic therapy [172–174], anti-inflammatory therapy [175–177], antioxidant therapy [178, 179], cell cycle therapy [180, 181], and hormonal therapy [180, 181]. Until a few years ago, the primary hurdle for developing an effective therapy for AD was an inability to increase redox potential inside mitochondria. Recently, various studies have reported potential breakthroughs in ameliorating mitochondria's antioxidant potential by using antioxidants that specifically target mitochondrial free radicals. These molecules offer several advantages, including preferential compartmentalization inside mitochondria, rapid neutralization of free radicals, and recycling of these compounds with no significant mitochondrial toxicity reported so far. However, research is still developing, and further studies are needed to see whether these compounds can be used in geriatric neurodegenerative diseases like AD.

### 4.1. Targeting Oxidative Stress by Antioxidants to Improve the Mitochondrial Dynamics in AD

Fortunately, selective antioxidant therapies demonstrate promise in ameliorating cognitive ability and restoring mitochondrial functioning. Henceforth, further investigation can provide a potential therapeutic protocol for the treatment of neurodegenerative diseases. Among the various therapeutic strategies, mitochondrial antioxidant therapy has been found to have a significant effect in amelioration of mitochondrial dysfunction, which results in restoration of mitochondrial dynamics without any appreciable adverse effects [182, 183]. Furthermore, encouraging results have been reported using the selective mitochondrial antioxidant treatment for restoring and rescuing mitochondria [183]. These studies used antioxidants viz acetyl-L-carnitine (ALCAR) and R-*α*lipoic acid (LA) in geriatric rats [184–186]. These findings were further supported by significant restoration in the function of hippocampal neurons. These neurons showed a smaller number of giant mitochondria when results were compared with the age-matched control group. On electron, microscopic examination, mitochondrion showed fewer ultrastructural abnormalities and lacked cistern rupture in the ALCAR/LA group. ALCAR/LA dietary supplementation in young rats indicated that more pronounced benefits could be achieved if these antioxidants are supplemented at an early age and early disease manifestation compared to supplementation made in the latter half. ALCAR/LA appears to be a potential therapeutic intervention in ameliorating cognitive dysfunctions in AD. Still, there is an urgent need to further investigate this therapeutic regimen in clinically controlled randomized clinical trials to see these drugs as clinical reality. Furthermore, the therapeutic role of antioxidants in ameliorating cognitive dysfunction in AD was observed in laboratory animal models of AD. In this study, vitamin E supplementation resulted in a significant reduction of *β* amyloid levels and restored cognitive function in the vitamin E-supplemented group compared to the control group [179]. Similarly, in another study, a tau pathology transgenic mouse model was used, and it was concluded that vitamin E administration could dissolve tau aggregates in the brain [178]. These studies suggest the direct effect of vitamin E on pathogenetic hotspots of AD. Furthermore, different antioxidant agents found to have protective action against AD are tabulated in Table 1.

#### 4.1.1. Mitochondrial-Targeted Antioxidants

The mitochondrion is called the cell's powerhouses as the inner membrane of mitochondria generates the driving force for ATP synthesis [207]. Accumulation of ATP molecules results in the generation of negative potential across the inner mitochondrial membrane. Researchers have used this negative potential to transfer lipophilic cations across the mitochondrial membrane and accumulate lipophilic cations within the mitochondrial matrix. Murphy and colleagues used this biological process to move reducing agents to the inner compartment of mitochondria, henceforth developing various mitochondrial-targeted antioxidants, which include MitoQ (a derivative of mitochondrial quinoline), MitoVitE (a derivative of mitochondrially targeted vitamin E) [223, 224], and MitoPBN (a derivative of *α*-phenyl-N-tert-butyl nitrone) [186]. These active lipophilic cations are attached with triphenylphosphonium vehicles, which help translocate these active molecules across mitochondrial lipid bilayer [184–186].

*MitoQ (Mitochondrially Targeted Ubiquinone):* MitoQ consists of two moieties which include oxidized mitoquinone and reduced mitoquinol; this adduct is attached to phosphonium cation [184]. After internalization into the mitochondrial membrane, the compound gets lodged in the inner membrane of mitochondria where it acts as an active donator of the hydrogen atom and henceforth prevents lipid peroxidation of mitochondrial lipid bilayer [225–227]. During this process, MitoQ moieties are transformed into semiubiquinone radicals, which disassociate into ubiquinone and ubiquinol [228]. Subsequently, ubiquinone is recycled back into ubiquinol, hence restoring its antioxidant function. The compound is found to accumulate selectively in mitochondria where it offers its role as a potent recyclable antioxidant, which eventually protects against AD (neuronal damage) [229]. Researchers across the globe have postulated that natural antioxidants are well tolerated in an AD mouse model and AD patients [178, 186, 228]. So, it can be postulated that MitoQ can offer a potential therapeutic option for treating AD in a mouse model and human patients.

*MitoVitE (Mitochondrially Targeted Vitamin E) and MitoPBN (Mitochondrially Targeted α-phenyl-N-tert-butyl nitrone)*: MitoVitE is derived from vitamin E specially targeted for improving mitochondrial dynamics as the compound is selectively accumulated inside mitochondria [207, 230]. Similarly, MitoPBN consists of [4-[4 (1,1-dimethyl ethyl)oxidoimino]-methyl]phenoxy]butyl] and triphenylphosphonium bromide and exhibits potential neuroprotective activity via removing free radicals generated and offers protection against free radicals' damage [186].

*Amino acid and peptide-based mitochondrial-targeted antioxidants*: these compounds are grouped under mitochondrially targeted antioxidants due to structural and conformational properties. The following amino acid and peptide-based mitochondrial-targeted antioxidants were developed to control oxidative damage induced by free radicals inside mitochondria (1) Dmt-D-Arg-Phe-Lys NH2 (SS-02), (2) Phe-D-Arg-Phe-Lys-NH2 (SS-20), (3) D-Arg-Dmt-Lys-Phe-NH2 (SS-31), and (4) Dmt-d-Arg-Phe-atnDAP-NH2 (SS-19) [207]. These compounds are highly permeable to the cell membrane and mitochondrial membrane. Studies have found 1000 folds higher levels of these compounds in the inner mitochondrial membrane than in the cytoplasm. The therapeutic action of these compounds has been attributed to reduced ROS generation, extensive mitochondrial uptake, inhibition of mitochondrial swallowing, mitochondrial depolarization, and prevented cytochrome C release. Hence, these compounds offer broad therapeutic potential for developing age-related diseases like AD [208].

### 4.2. Maintaining the Mitochondrial Bioenergetic Performance

In AD, mitochondrial function and architecture are altered [138, 231, 232], hence searching for those classes of compound that can restore functional and structural capabilities of mitochondria began in the latter half of the 19^th^ century. Researchers have identified various compounds that can present an effective therapy for AD treatment. This section will discuss multiple therapeutic regimens that help maintain mitochondrial bioenergetic performance.

#### 4.2.1. Cellular Therapy

Cellular therapy is being evaluated in laboratory animal models of AD [233, 234]. Neurons and other associated structures of nervous tissue have been successfully generated by using embryonic stem cells (ESCs) and induced pluripotent stem cells (iPSCs), followed by successful transplantation to laboratory animal models for treatment of neurodegenerative diseases, including AD [235]. The transplantation resulted in a significant increase in mtDNA (mitochondrial DNA), mRNA (messenger RNA), and associated proteins required for mitochondrial biogenesis and mitochondrial fission. Furthermore, this therapy protocol resulted in significant restoration of mitochondrial functioning and increased levels of morphologically well-structured mitochondria in nervous tissue, which was reflected in the amelioration of cognitive functioning and clinical improvement in laboratory animal models [236].

#### 4.2.2. Targeting the Inflammasome

Among the various inflammasomes, the NLRP3 category of inflammasomes plays a pivotal role in the deposition of A*β* aggregates and hence has an important role in the pathogenesis of AD [237–239]. Based on these assumptions, researchers conducted several studies using micro inhibitors of NLRP3 inflammasome and found that these molecules ameliorate AD pathology [240]. To support the inflammasome' function in the AD pathogenesis, researchers postulated that brain-penetrant anti-inflammatory neurotrophic drug, CAD-31, which ameliorates cognitive abilities, regenerates synaptic loss, helps in the removal of A*β* aggregates, and restores bioenergetics of mitochondria [138, 231–244].

#### 4.2.3. Targeting the Proteasome

A plethora of studies have found an ameliorative role of proteasome activation in neurodegenerative diseases like AD [245–248]. Different strategies are used for proteasome activation, an efficient pathway involved in the genesis of protein kinase A and cyclic AMP (cAMP), which increases proteasome function, subsequently decreasing tau aggregate levels and improving cognitive function functioning [247]. In addition to this, other strategies under investigation for enhancing the activity of proteasome functioning include inhibition of USP14 that inhibits processing of proteasome employing pyrazolone and inhibition of the deubiquitinating enzyme that causes proteolysis of the proteasome [249, 250].

#### 4.2.4. Targeting mtDNA

With the progression of age, there occurs accumulation of mutations in the mitochondrion [251] which pose a higher risk for the pathogenesis of AD [252].To slow down the pace of these mutations and correct the mutations, various molecular techniques are proposed, which include (i) the clustered regularly interspaced short palindromic repeats (CRISPR)/associated protein 9 (CRISPR/Cas9) technology is presently being used to correct the deleterious mutations in the mitochondrial genome [253]. The technique uses mitoCas9, explicitly targeting the mitochondrial genome without affecting genomic DNA. (ii) Transcription activator-like effector nuclease (TALEN) is a novel technique that specifically targets mutated mtDNA and effectively causes cleavage of these mutated fragments, which results in a reduction in levels of potential pathogenic mtDNAs, hence retarding the progression of diseases like AD [254]. The technique has been successfully used to correct mtDNA mutations in respiratory diseases and correct enzyme dynamics involved in oxidative phosphorylation [255].

#### 4.2.5. Targeting Mitochondrial Cholesterol

**Figure 4:**
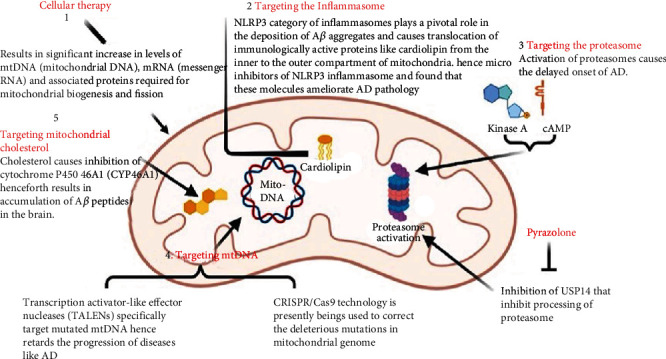
Maintaining the mitochondrial bioenergetic performance. Various means can be employed to maintain the mitochondrial bioenergetic performance including cellular therapy, targeting inflammasomes, proteosome, mitochondrial cholesterol, and mtDNA.

Deposition of cholesterol in mitochondrial membrane results in reduced flexibility and fluidity of membrane structure [256]. To support this proposition, studies have found a direct relationship between neurodegenerative diseases and changes in mitochondrial lipid composition [257]. Recently, studies in the APP23 AD mouse model have found that cholesterol causes inhibition of cytochrome P450 46A1 (CYP46A1), henceforth resulting in A*β* peptide accumulation in the brain [258, 259] (Figure 4).

*Biologics.* Manipulating mitochondrion function via selective genomic expression of mtDNA offers crucial therapeutic hope for patients suffering from neurodegenerative diseases like AD. To increase ATP levels from the mitochondrion, mitochondrial transcription factor A (TFAM) has been engineered in such a way that this engineered molecule passes readily across the cellular and mitochondrial membrane and causes selective genomic expression, which causes reduction in levels of A*β* in 3xTg-AD mice, increases levels of the transthyretin (a potent inhibitor of A*β* aggregate) and causes a reduction in levels of mitochondrial mutations [260]. Laboratory animal studies have revealed that recombinant-human Transcription Factor A Mitochondrial protein (rhTFAM) results in restoring cognitive function in a laboratory model of AD compared to the control group [261–263].

### 4.3. Improvement in Proper Transportation and Mitophagy in AD

Of the particular interest in AD, extensive axonal degeneration occurs due to the accumulation of defective mitochondrion. The scientific community is trying to explore how and why mitochondrial trafficking activates the pathological course of AD [264]. In neurons, most of the mitochondria are sessile. In contrast, only a tiny portion of mitochondria move in neurons' upward and downward direction as per the energy requirements [265]. In neurons, two classes of proteins cause directional movement of mitochondria. These are kinesin and dynein. Kinesin regulates upward movement, while dynein regulates the downward trend of mitoplasts [123]. Most therapeutic regimens have targeted these two pathways to improve mitochondria's proper transportation. To ensure smooth mobility of mitochondria inside neurons, the energy source (ATP) and regulators of mitochondrial transport should be present in the vicinity of the mitochondrion [266]. Recent studies have shown that pharmacological intervention to increase cellular energy sensor AMP-activated protein kinase (AMPK) increases the upward movement of mitochondria along the axon and increases the branching of axons [267]. Furthermore, hypoxia in experimental animal studies was found to enhance mitochondrial transportation through induction of hypoxia-upregulated mitochondrial movement regulator (HUMMR) [268, 269]. HUMMER interacts with adaptor and docking complexes and enhances anterograde transportation of mitochondria. These studies further added that genetic ablation of HUMMR leads to an increased mitochondrial percentage moving in the retrograde direction, and subsequently, a lesser number of mitochondria moving in the anterograde direction [270]. Additionally, when neurons were subjected to hypoxic and hypoglycemic microenvironmental conditions, these neurons transported mitochondrion from neighboring astrocytes and released defective mitochondria, which restored their bioenergetic functions [271]. These studies concluded the therapeutic role of hypoxia and genetic manipulation of the HUMMR gene for patients suffering from impaired mitochondrial transport in AD. Similarly, parental administration of mitochondria harvested from the liver of young mice resulted in the restoration of bioenergetic functions, amelioration of oxidative damage, and restoration of cognitive and motor function in aged mice [272]. Mitochondrial-targeted antioxidant SS31 restored mitochondrial mobility in a rat model of AD. SS31 peptide is directed towards the inner mitochondrial membrane due to attraction between the positive charge of SS31 molecule and negative charge of cardiolipin molecules located on the inner mitochondrial membrane. In addition to this, SS31 molecule acts as an efficient scavenger of ROS and inhibits the opening of mitochondrial pores, hence swelling of mitochondria. These studies suggest that the SS31 molecule should be studied as a potential drug to treat AD [270]. Furthermore, the antioxidant SS31 peptide reduced levels of mitochondrial fission proteins, increased mitophagy [124], and restored mitochondrial trafficking deficit [273]. Therefore, drugs that promote mitophagy help regenerate dysfunctional mitochondria [274]. Various rate-limiting steps in mitophagy have been identified; for instance, PINKI (protein kinase 1) is a critical molecule in the mitophagy pathway [275]. Henceforth, those drugs that accelerate these mitophagy pathways appear promising for many neurodegenerative diseases, including AD. To exemplify, autophagy inducers like rapamycin help in the prevention of mitochondrial fission in the rat model of AD [276]. Similarly, studies have found that nuclear receptors peroxisome proliferator-activated receptors gamma (PPAR*γ*) and PGC1-alpha play a pivotal role in mitochondrial biogenesis [277]. Interestingly, both PPAR*γ* and PGC1-alpha are significantly reduced in AD; hence, drugs that cause the promotion of mitochondrial biogenesis through activation of PPAR*γ* and PGC1-alpha can emerge as potential therapeutic targets for the treatment of mitochondrial dysfunctions in AD [276]. For instance, the use of drugs like thiazolidinediones that cause activation of these molecules has improved cognitive activity in AD mice and clinical cases with mild degrees of AD [278] (Table 2).

More recently, researchers have devised a novel technology of mitochondrial transplantation as an effective therapeutic regimen to restore cellular function in various animal models of human diseases. The technique was first reported by Katrangi and colleagues when coincubated xenogenic mitochondria derived from mice with human cells devoid of mitochondria [292]. They found that human cells incorporated xenogenic mitochondria, restoring aerobic respiration. However, recently, this strategy has been employed as a therapeutic option for treating different neurodegenerative diseases in a wide range of experimental animals. For instance, mitochondria labeled with the marker (green fluorescent protein) obtained from leg muscle were injected with a damaged spinal cord in an animal model. After two weeks, these mitochondria were found in nervous tissue and motor neurons, producing some neuroprotective effects [293]. Although the current strategy has been translated to clinical cases for the treatment of cardiac and neurodegenerative diseases; however, the effectiveness of the current strategy in age-related neurodegenerative diseases is in its early stages, and to standardize this strategy, there is an urgent need for more studies to evaluate the neuroprotective role of mitochondria transplantation and associated risk involved.

Mitochondria isolated from patients with AD were found to have altered calcium homeostasis, which could cause the opening of mitochondrial permeability transition pores (mPTP) [294]. Altered calcium homeostasis causes an elevation in levels of CypD, which serves as a structural element for the synthesis of mPTP and triggers the opening of mPTP. Studies in the knockout model of the CypD gene showed reduced permeability of mPTP and higher efficiency of mitochondria to tolerate calcium imbalance [295]. Furthermore, studies have found that inhibition of CypD improves mitochondrial function; hence, inhibitors of CypD can serve as potential drugs for the prevention and treatment of AD [296]. Furthermore, laboratory animal models for AD treated with CsA showed improved cognitive function and motor activity attributed to enhanced mitochondrial transmembrane potential and activation of superoxide dismutase activity [297]. In this realm, researchers have found some compounds that act on several pathways of mitochondrial dysfunction. For instance, in response to free radicals, nuclear factor E2-related factor 2 (Nrf2) is translocated into the nucleus from the cytoplasmic matrix, which results in increased expression of genes associated with antioxidant defense; Nrf2 induces several beneficial modifications in mitochondrial architecture and dynamics, which are of particular interest for the proper functioning of mitochondria under hostile conditions [298]. Of particular interest, the dietary molecule sulforaphane (SFN) acts as a potent activator of Nrf2 and can be processed as a dietary nutraceutical against AD [299].

### 4.4. Improvement in Mitochondrial Health by a Change in Lifestyle

#### 4.4.1. Calorie Restrictions (CR)

CR leads to de novo synthesis of an efficient mitochondrion with an efficient ATP/ROS ratio, leading to diminished free radicals/ATP-generated production. The decreased levels of free radicals by CR are through activation of sirtuin-3-dependent superoxide dismutase 2 [300–302]. In addition, CR promotes mitophagy of defective mitochondria [303]. In addition to mitophagy, CR promotes the synthesis of the new mitochondrion and restores the function of various genes, which lose their expression with aging. To special interest, these genes are primarily associated with mitogenesis. If these defective mitochondria are not removed by mitophagy, there is an accumulation of defective mitoplast that promotes the accumulation of A*β* aggregate [304]. In several experimental studies, CR was found to regulate/modulate inflammatory pathways; for instance, CR was found to normalize concentrations of proinflammatory mediators (TNF-*α* and IL-6) in geriatric mice to youthful profile levels [305]. There are various pathways by which fasting exerts its beneficial action; for instance, an elegant study published recently found CR results in the accumulation of ketone bodies which subsequently downregulates inflammatory pathways by blocking inflammasomes and producing cytokines from monocytes. In the context of neurological diseases, CR has specifically demonstrated important immune-inflammatory modulatory actions; in laboratory animal studies, CR has demonstrated modulation of microglia activation and restoration of cognitive dysfunction in the AD rat model [306, 307].

*(1) Calorie Restriction Mimetic*. Given the health-promoting role of CR in AD, there are a vast number of strategies currently under investigation to generate and characterize new compounds collectively called CR mimetics. These compounds stimulate the action of CR on various pathways and biochemical reactions without any harmful effects.

- NAD^+^ Precursors

NAD^+^ precursor supplementation causes downregulation of inflammatory pathways and promotes phagocytosis of A*β* aggregate [308, 309] and, hence, inhibits AD progression. Interestingly with the progression of age, levels of NAD^+^ decrease, which cause hyperactivation of the above-mentioned pathways suggesting one of the potential pathogenetic pathways for the progression of AD [310, 311]. Recently, results from preclinical studies have proposed a novel strategy called “turning up of the NAD^+^-mitophagy axis” [167]. Several therapeutic strategies have been employed for turning up strategies; one of the strategies involves restoration of activity of SIRT3 and SIRT1 by direct increasing levels of NAD^+^. In preclinical studies, using 3xTgAD mice supplementation with nicotinamide, a precursor of NAD^+^, resulted in a significant reduction in amyloid plaques. Moreover, treatment of APP mutant transgenic mice with nicotinamide riboside resulted in a turning up of the NAD^+^-mitophagy axis. These preliminary studies support hypotheses that increasing levels of NAD^+^ can be beneficial for patients with AD. Although the mechanism that governs the amelioration of AD pathology following supplementation with NAD^+^/NAD^+^ precursor remains poorly understood, researchers have formulated some tentative ideas. The well-accepted hypothesis postulates that increased levels of NAD^+^ cause microglial-dependent elimination of extracellular A*β* plaques and simultaneously cause inhibition of NLRP3 inflammasome [168].

(b) Resveratrol

Encouraging results with the use of resveratrol have been observed against neuroinflammation and neuroinflammatory diseases mediated through AMPK and Sirt1 activation. In vitro coculture experiments have found that resveratrol causes quiescence of microglia upon exposure to A*β* aggregate, inhibiting the progression of neuroinflammation and subsequently protecting the death of neurons [312, 313].

(c) Metformin

It is a standard drug used to treat diabetes mellitus; cumulative evidence suggests that the drug acts on a different biological pathway involved in the aging process. Hence, it can be postulated that drugs can be beneficial against geriatric diseases like AD [314, 315]. Mainly, metformin has been found to restrict the recruitment of immune cells and downregulates inflammatory pathways in the central nervous system [316]. The therapeutic action of metformin in AD is still controversial. However, it causes a reduction in levels of microglia and astrocyte, downregulates expression of NF-kB signaling, inhibits phosphorylation of tau protein, and causes effectively clearance of toxic proteins accumulated in AD [317, 318].

(d) Spermidine

Of particular interest, spermidine helps renew mitochondrial function by promoting old and damaged mitochondrion mitophagy and mitogenesis of new and efficient mitochondrion [319, 320]. Spermidine exerts an anti-inflammatory action through epigenetic inhibition of lymphocyte migration; the mechanism involves hypermethylation of the *Itgal* gene, which is involved in the biosynthesis of adhesion molecule LFA-1 [321]. Spermidine induces differential expression of proinflammatory cytokines, including enhanced production of anti-inflammatory cytokines and reduced proinflammatory cytokines [322]. Hence, it can be proposed that spermidine exerts its effect through differential expression of genes and mediators involved in the neuroinflammatory cascade.

#### 4.4.2. Different Kinds of Diets

Several published and unpublished studies have established a relationship between diet and AD [323]. Various ingredients in diet like polyunsaturated fatty acids, vitamins, Mediterranean diet, ingredients in fruits and vegetables, and active principles derived from medicinal plants like curcumin have been reported to have both prophylactic and therapeutic activity against AD [324–326].

*(1) Mediterranean Diet*. People living around the Mediterranean Sea consume a high number of fresh fruits, fresh vegetables, and grains. These people use olive oil as the major source of fat [327, 328]. Both epidemiological and clinical studies have found a relatively lower incidence of cognitive decline and AD in people living around the Mediterranean Sea [210]. On further investigation, researchers have attributed the higher number of polyphenols like flavonols, resveratrol, and omega-three fatty acids in the Mediterranean diet, which improves the cognitive functioning of the geriatric population, henceforth offering protection against neurodegenerative diseases like AD [211, 329, 330].

*(2) Ketogenic Diet*. Preclinical and clinical studies have found a region-specific decrease in glucose utilization in patients with AD while ketone body utilization remains unaffected [213]. Supplementation of ketogenic components in diet augments the supply of alternative fuel to the brain [331]. Although the mechanism underlying ameliorative effects of cognitive function in AD patients is yet to be established, researchers have postulated that the inclusion of ketogenic diet results in the normalization of energy balance in AD patients [332].

#### 4.4.3. Physical Exercises

**Figure 5:**
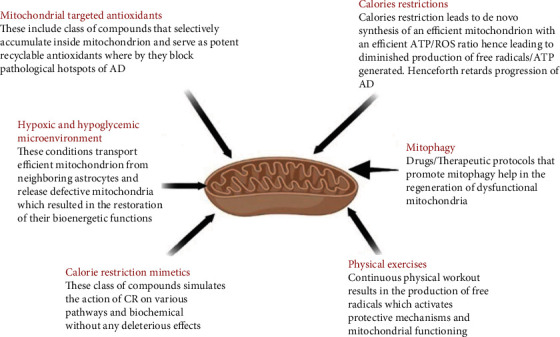
Therapeutic strategies for the improvement of mitochondrial dynamics.

Several preclinical and clinical studies have reported the significant effect of various nonpharmacological interventions on ameliorating cognitive symptoms in AD patients [330–335]. Continuous physical workout causes the generation of free radicals, which activates protective mechanisms and mitochondrial functioning [336]. In animal model studies, exercise was found to reduce A*β* plaques in the brain's hippocampus region [337]. Physical activity in the AD animal model ameliorated oxidative stress, helped insulin resistance, and reduced cholesterol levels [337]. Similarly, studies have found the pivotal role of exercise in the induction of vascularization, neurogenesis, synaptogenesis, and angiogenesis [338, 339]. Added advantages are observed in combining exercise and CR in restoring neurological function, cognitive function, and improvement of memory deficits [340]. However, some studies have reported no significant beneficial effects of exercise on cognitive function in diseases like depression and AD [339]. Furthermore, many randomized controlled trials (RCT) have found some benefits of physical exercise in individuals with mild to moderate degrees of AD, while no clinical benefits were observed in severe and chronic AD [341, 342]. Similarly, many studies have found significant efficacy of combinational nonpharmacological interventions compared to a single invention in ameliorating cognitive deficit in AD patients [343]. A pioneer RCT was conducted to investigate the effect of multicomponent cognitive intervention in AD patients with mild derangement in cognitive function. The study reported no significant improvement in mental activity in the intervention group compared to the control group [344]. This was followed by another long-term (12 months) multicomponent cognitive interventional RCT study in patients with moderate AD. They reported no change in cognitive function in the intervention group, while there was deterioration in mental activity in the control group [345]. From these studies, it can be postulated that multicomponent cognitive intervention has no significant effect in ameliorating cognitive deficit in clinical cases with mild to moderate AD. But some studies support the role of exercise in the amelioration of cognitive deficit in AD, so it can be postulated that there exists a lack of consensus among different researchers regarding the role of exercise in the resolution of cognitive function in AD patients [346, 347]. Recently, a meta-analysis postulated that regular walking and cycling improve cognitive abilities in AD patients with mild to moderate derangement. Strength training effectively restores motor functions, which indirectly reduces the risk of developing AD [348]. Furthermore, this meta-analysis reported the preventive effects of physical exercise against AD. In addition, laboratory animal studies have found improvement in motor function, cognitive function, and reduction in A*β* aggregate in mice under regular physical exercise [349, 350]. The same study found that exercise causes increased blood flow to critical areas of the brain, which induces neurogenesis in the hippocampus. Following these results, many authors have proposed that physical exercise causes a reduction in cholesterol levels, restoration of insulin sensitivity, scavenging free radicals' neurogenesis, and synaptogenesis, which indirectly promotes mitophagy of defective mitochondrion and induces *de novo* synthesis of the new mitochondrion. Based on these findings, it can be proposed that there is a need for extensive studies involving large patient sizes and a need to establish a standard protocol to procure the benefits of physical exercise in patients with AD and other geriatric neurodegenerative diseases (Figure 5).

## 5. Conclusion

Although mitochondrial dysfunction is a typical indication of Alzheimer's disease, it is unclear whether the cellular systems that maintain mitochondrial integrity malfunction, aggravating mitochondrial pathology. Different levels of vigilance and preventive methods are used to reduce mitochondrial damage and efficiently destroy faulty mitochondria to maintain the mitochondrial equilibrium. The form and function of mitochondria are regulated by mitochondrial fusion and fission. In contrast, mitochondrial transit holds mitochondrial dispersion and transports old and damaged mitochondria from distant axons and synapses to the soma for lysosomal destruction. As the fundamental mechanisms of mitochondrial quality control, several critical properties of mitochondria work in tandem with mitophagy. According to the findings, mitochondrial viability and function are managed by mitochondrial fusion, fission, transport, and mitophagy, forming a complex, dynamic, and reciprocal interaction network. According to growing evidence, AD brains have disrupted mitochondrial dynamics and aberrant mitophagy, which may interfere with mitochondrial quality control directly or indirectly [351].

Further research into these processes might help us better understand mitochondrial malfunction in Alzheimer's disease. Given the ability to improve some phenotypes by manipulating genes that regulate mitophagy, there is reason to believe that attempting to subvert mitochondrial dynamics, motility unilaterally, and mitophagy will enhance mitochondrial surveillance mechanisms and decrease the neuropathology of Alzheimer's disease, feasibly leading to new treatment strategies. Many investigations on phosphorylated tau, A*β*, inflammatory reactions, synaptic and mitochondrial activity in the progression of the disease, and pathogenesis have aided researchers in their understanding of AD. Therapeutic techniques have been created and assessed in many trials using postmortem AD brains, AD mouse models, cell cultures, and blood-based indicators. Clinical experiments in the past and present have had mixed results. Future studies focus on synaptic alterations that are crucial to disease development. Hence, AD is a complex disease that warrants more exploration due to a combination of synapse loss and mitochondrial deficits and faulty mitophagy induced by A*β*, tau, and mitochondrial and synaptic problems.

## Figures and Tables

**Table 1:** The antioxidants (diet/drug/active principle) having a protective effect against AD with their postulated mechanism of action.

Diet/drug/active principle	Type of study/animal/cell model	Mechanism of action	References
Acetyl-L-carnitine (ALCAR) and R-*α* lipoic acid (LA)	Geriatric rats	Restoration of cognitive abilities, reduction in oxidative stress markers, and rescuing mitochondrial dynamics in rat parenchymal stoma	[183–186]
Coenzyme Q 10	Preclinical studies	Coenzyme Q 10 specifically targets mitochondrial free radicals, enhances ATP synthesis, and ameliorates mitochondrial dysfunction, henceforth causing improvement in cognitive function. The laboratory animal model (ICV-STZ) of AD supplemented with CoQ10 resulted in restoration of choline acetyl transferase enzymatic activity	[187][188]
	Clinical studies	In clinical studies, no improvement was reported in clinical cases supplemented with CoQ10	[189]
Creatine	Preclinical studies	In preclinical studies, creatine supplementation was reported to restore motor neuron activity, ameliorates mitochondrial dysfunction, and modulates amyloid-beta-induced cell death	[190]
	Clinical studies	Reduced blood levels of 8-hydroxy-2-deoxyguanosine which is a biomarker of oxidative damage. Henceforth, amelioration of oxidative stress causes restoration of neurological functions in various neurodegenerative diseases	[191]
Idebenone	Preclinical studies	Preclinical studies have found Idebenone supplementation inhibits amyloid-beta-induced neurotoxicity	[192]
	Clinical studies	In clinical studies, Idebenone was reported to cause improvement in cognitive and molecular score in Alzheimer's disease	[193]
Latrepirdine	Preclinical studies	Latrepirdine acts on various pathways that induce mitochondrial dysfunction	[194]
	Clinical studies	Latrepirdine causes improvement in clinical score of patients with AD	[195]
Triterpenoids	Preclinical studies	Triterpenoids were found to cause activation of the Nrf2/ARE signaling pathway which helps in protection of neurons against various types of insults	[196]
MitoTEMPOL (4-hydroxy-2,2,6,6,-tetramethylpiperidine- 1-oxyl)	Preclinical studies	Compound acts on the mitochondrial antioxidant pathway and ameliorated oxidative damage, hence restoring mitochondrial function	[197]
SS (Szeto-Schiller) peptides	Preclinical studies	These peptides act on multiple pathways, for instance, mitochondrial ROS generation, restore mitochondrial swelling, and henceforth inhibit releases of mitochondrial contents	[198]
Methylene blue	Preclinical studies	Methylene blue inhibits pathological pathways in AD	[199]
Curcumin	p25 transgenic mouse model	Reduced oxidative damage and A*β* deposits	[200]
Melatonin	Tg2576 mice	Decreases the levels of A*β* and protein nitration, henceforth increasing lifespan of laboratory animals	[201]
Dismutase catalase mimetics	AD transgenic mice	Prevented cataracts in AD mice	[202]
Diets supplemented with vitamin E	AD patients	Amelioration of AD symptoms	[203]
Combined supplementation of vitamin E and vitamin C	Elderly patients	Therapeutic and prophylactic action against AD	[204]
Cholinesterase inhibition and huperzine A (an antioxidant)	AD patients	Restoration of cognitive functions and decreased levels of 21 amyloid proteins	[205]
Synthetic mitochondrial antioxidants	*Saccharomyces cerevisiae* lacking CuZn-superoxide dismutase	Increased lifespan and reproductive rate of yeast	[206]
SS-31	Isolated mitochondria	Molecule becomes localized at ROS production	[207]
SS tetrapeptides	Neuronal cell lines	Protection against mitochondrial dysfunction and apoptosis	[208]
N-[4-(Octa-O-acetyllactobionamidomethylene) benzylidene]-N-[1,1-dimethyl-2-(N-octanoyl) amido]-ethylamine N-oxide (LPBNAH)	Rotifers	Neuroprotective activity and antagonizes oxidotoxicity	[209]
MitoPBN, MitoVitE, MitoQ	Cell lines, laboratory animal model, and clinical patients	Preferential compartmentization inside mitochondria, rapid neutralization of free radical, recycling of these compounds with no significant mitochondrial toxicity	[184–186]
Mediterranean diet	Epidemiological studies and clinical trials	Polyphenols, carotenoids, and sulfur compounds act as neuroprotective	[210]
Dietary polyphenolic compounds	In vitro studies	Mitigate microglial-mediated neuroinflammation	[211]
Resveratrol	Rodent models	Amyloid deposition is reduced and causes dephosphorylation of tau-hyperphosphorylation proteins	[212]
Ketogenic diets	Epidemiological studies and clinical trials	Supplies alternative source of energy to neuronal tissue in later life	[213]
Souvenaid®	Clinical trials	These classes of molecules improve synaptic function by supplying precursors at nerve ends	[214]
Axona®	Clinical trials	Provides bodies alternative energy supply to neurons	
CerefolinNAC®	Clinical trials	Ameliorates oxidative stress	
Epigenetic diet	Clinical trials and experimental studies	These type of diets results in DNA methylation and posttranslational modification in histone proteins	[215]
Huperzine A	Clinical trials and experimental studies	Act as an acetylcholinesterase inhibitor (AchEI), reduces amyloid plaque production, and inhibits cell death by modulating neuronal iron content in animal models of AD	[216] [217]
Gingko biloba	Clinical trials	Slows progression and ameliorates mild cognitive impairment in AD	[218]
Cineole	Beta amyloid treated PC12 cells	Reduces inflammation and oxidative stress	[219]
Coconut oil	Epidemiological studies	The postulated mechanism for the effect is the content of caprylic acid that restores brain function	[220]
Fish oil	Epidemiological studies	High content of apolipoprotein E which is neuroprotective in nature	[221]
Thymoquinone	Beta amyloid treated PC12 cells	Inhibition of mitochondrial dysfunction and oxidative stress	[222]
Organic selenium	Experimental studies	Acts as an antioxidants and helps in regeneration of neurons	[223]

**Table 2:** Nontoxic mitophagy inducers used as drug candidates for AD.

S. No.	Nontoxic inducer of mitophagy	Mechanism of action	Clinical trial on AD	References
1.	Metformin (Met)	Facilitates Parkin-mediated mitophagy, induces AMPK-independent and SIRT1-mediated pathway	Yes	[279]
2.	Nicotinamide mononucleotide (NMN)	Enhanced PINK-1, PDR-1, or DCT-1-dependent pathways	Yes	[280]
3.	Resveratrol (Res)	A stilbenoid activates AMPK and SIRT1 deacetylase activity	Yes	[281]
4.	Actinonin	An antibacterial agent promotes mitochondrial fission and enhances kinase activity of PINK1	No	[282]
5.	Rapamycin	Induces genes (*PINK1*, *ULK1*, *AMBRA1*, and *PARK1N*) promoting mitophagy and (*DRP1* and *FIS1*) mitochondrial fission	No	[283]
6.	Urolithin A	A gut microbiome-derived natural compound activated mitophagy and reduces inflammation	No	[284]
7.	Fisetin	Blocks NLRP3 inflammasome activation via promoting mitophagy, reduces neuroinflammation, and restores cognitive impairment	No	[285]
8.	Spermidine	Induces PINK1-PDR1-dependent mitophagy pathway	No	[286]
9.	Ciclopirox olamine	Induces mitophagy by dissipation of mitochondrial membrane and DRP-1-dependent mitochondrial fragmentation	No	[282]
10.	Nicotinamide (NAM)	Induces mitophagy by increasing the ratio of NAD^+^/NADH and SIRT1 activation	No	[287]
11.	Deferiprone (DFP)	An iron chelator induces mitophagy by a Parkin-independent pathway	No	[288]
12.	Kinetin triphosphate (KTP)	PINK1 activity is activated, blocks mitochondrial motility, and inhibits apoptosis in human neurons	No	[289]
13.	Pifithrin-a	Specific p53 inhibitor improves mitochondrial dysfunction, protects Parkin-mediated mitophagy	No	[290]
14.	Kaempferol and rhapontigenin	The survival of glutamatergic and cholinergic neurons was elevated, enhanced animal memory, and revoke tau and amyloid pathogenesis	No	[291]

## Data Availability

Data supporting this review are from previously reported studies, which have been cited.
